# Decoding Effector‐Specific Parametric Grip‐Force Anticipation From fMRI‐Data

**DOI:** 10.1002/hbm.70441

**Published:** 2025-12-30

**Authors:** Guido Caccialupi, Timo Torsten Schmidt, Felix Blankenburg

**Affiliations:** ^1^ Neurocomputation and Neuroimaging Unit (NNU), Freie Universität Berlin Berlin Germany; ^2^ Berlin School of Mind and Brain Humboldt Universität Zu Berlin Berlin Germany

**Keywords:** effector‐specific representation, fMRI, grip‐force, motor planning, motor preparation, MVPA, prospective working memory

## Abstract

Planning motor‐actions involves the neuronal representation of key parameters such as force and timing prior to execution. Functional magnetic resonance imaging (fMRI) studies have shown that activity in premotor and parietal areas covaries with these parameters during motor‐preparation. While previous research has demonstrated that parametric codes reflect graded grip‐force intensities before and after their transformation into motor‐codes, it remains unclear whether these representations are encoded in effector‐specific brain‐regions. To address this, we conducted an fMRI‐study using a delayed grip‐force task in which participants prepared one of four force‐intensities with either their right or left cued‐hand, with the hand to‐be‐used being switched in 50% of the trials midway through the delay. Using time‐resolved multivoxel pattern analysis (MVPA) with a searchlight approach, we identified brain‐regions encoding anticipated grip‐force intensities of the cued‐hand across the two 6‐s delay‐periods. In addition, cross‐decoding analyses tested whether force‐intensities were represented in an effector‐specific or effector‐independent format. We found above‐chance decoding in two lateralized networks: the contralateral intraparietal sulcus (r−/l‐IPS), as well as the lateral occipitotemporal cortex (r−/l‐LOTC) during the first, and the contralateral primary motor cortices (r−/l‐M1) during the second delay. These results indicate effector‐specific coding of anticipated grip‐force intensities, which is revealed by systematic lateralization of decoding‐accuracy depending on the hand to‐be‐used. Cross‐decoding corroborated effector‐specific representation in these regions. Together, our results show that contralateral IPS and LOTCs encode effector‐specific parametric information prior to M1s, likely reflecting a transformation process in which the intended grip‐force intensity is selected, maintained, and then converted into detailed movement‐plans.

## Graphical abstract

In an fMRI study, we used a delayed grip‐force task and time‐resolved MVPA to test where anticipated grip‐force intensities are parametrically encoded, whether these are represented in effector‐specific brain regions, and how these representations are transformed across two 6‐s delay periods. During the first delay, contralateral intraparietal sulci (l‐IPS, r‐IPS) and extrastriate body areas (l‐EBA, r‐EBA) encoded the intended grip‐force, which was later transformed into motor codes in contralateral primary motor cortices (l‐M1, r‐M1). Systematic above‐chance decoding in contralateral regions of the to‐be‐used hand indicates effector‐specific coding, corroborated by cross‐decoding.

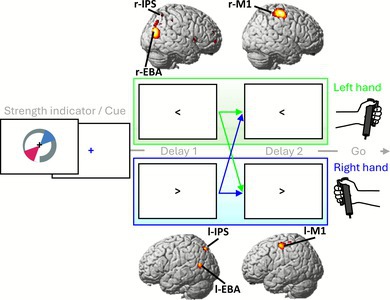

## 1 Introduction

Motor planning is a complex neural process that underpins the ability to anticipate, represent, and prepare parameters of upcoming actions prior to execution. This process involves selecting the appropriate effector (i.e., the body part with which an action is to be performed; Gallivan, McLean, Smith, and Culham 2011; Esmeyer et al. 2025), maintaining relevant motor parameters in working memory (WM), and transforming abstract motor goals into executable movement plans (Boettcher et al. 2021). While neuroimaging research has extensively investigated the neural correlates of action selection (Soon et al. 2008, 2013; Bode and Haynes 2009) and motor execution (Andrushko et al. 2021; Ruiz et al. 2024), the intermediate processes linking these stages—specifically, the effector‐specific encoding of motor goals and their transformation into movement plans—remain less understood (Kim et al. 2021; Ruiz et al. 2024). Recent studies using electroencephalography (EEG) (Van Ede 2020; Henderson et al. 2022) and functional magnetic resonance imaging (fMRI) (Caccialupi et al. 2025) have begun to examine how the human brain encodes, maintains, and translates intended motor outputs into movement plans before execution (for recent work on intracranial recording in non‐human primates, see Intveld et al. 2018). These studies distinguish different phases of cue perception, WM, movement planning, and execution, providing first insights into the sequence and transformation of these processes.

Numerous neurophysiological studies in animals have identified preparatory signals reflecting planned actions in the dorsal premotor cortex (PMd; Cisek and Kalaska 2004, Cisek and Kalaska 2010; Hoshi and Tanji 2006), supplementary motor area (SMA; Hoshi and Tanji 2000), and posterior parietal cortex (PPC; Cui and Andersen 2007, 2011; Andersen and Cui 2009). Complementary neuroimaging studies in humans, using delayed‐response paradigms (e.g., Hamilton and Grafton 1993), have consistently shown that premotor areas—particularly the PMd—are involved in planning upcoming movements (e.g., Wong and Haith 2017). For instance, univariate fMRI analyses in delayed grip‐force tasks have demonstrated that BOLD signal amplitude in the PMd and intraparietal sulcus (IPS) covaries with anticipated grip‐force intensity, indicating parametric encoding of movement parameters prior to execution (Cole and Rotella 2002; Chouinard et al. 2005; Nowak et al. 2009; Van Nuenen et al. 2012; Mizuguchi et al. 2014). Recent fMRI multivoxel pattern analysis (MVPA) studies have further shown that above‐chance decoding in regions of an extended parieto‐frontal network—including parietal, premotor, and motor cortices—can predict various movement properties, such as movement sequences (Yokoi and Diedrichsen 2019; Ariani et al. 2021, 2022), the to‐be‐used effector (Gallivan, McLean, Smith, and Culham 2011; Gallivan, Chapman, et al. 2013; Leoné et al. 2014; Turella et al. 2020), kinematics of grip types (Gallivan, McLean, Smith, and Culham 2011; Ariani et al. 2015; Ruiz et al. 2024), and anticipated grip‐force intensity (Caccialupi et al. 2025). These findings further corroborate the involvement of parieto‐frontal networks in encoding movement properties and anticipating motor parameters.

Building on these findings, recent fMRI MVPA studies have revealed that information about the outcome of an intended action—or its “goal” (Cattaneo et al. 2009; Gallivan and Culham 2015; Turella et al. 2020)—can be decoded from the posterior parietal cortex (PPC) prior to movement planning (Gallivan, McLean, et al. 2013; Ariani et al. 2018). This finding aligns with a two‐stage model in which the intended action is first selected and then motor movements are specified and prepared for execution (Boettcher et al. 2021). Consistent with this framework, the intraparietal sulcus (IPS) has been found to support the transformation of abstract action intentions into motor codes during early stages of motor planning (Caccialupi et al. 2025). Although both effector‐specific and effector‐independent information has been decoded from this region (Ariani et al. 2015; Gallivan et al. 2015), a more posterior section of the IPS (pIPS) has been found to mainly support effector‐specific encoding during motor planning (Ariani et al. 2015). Similarly, high‐level sensory regions within the lateral occipitotemporal cortex (LOTC) have been associated with processing body‐related aspects of to‐be‐performed actions (Lingnau and Downing 2015). For instance, the fusiform face area (FFA) has often been found to encode information about specific body parts in tasks that do not involve direct control of forthcoming motor movements, whereas the extrastriate body area (EBA) has been primarily implicated in anticipatory motor processes, encoding information about the intended motor action in an effector‐specific format (Gallivan, McLean, et al. 2013). While the EBA is traditionally associated with the visual perception of body parts, it shows lateralized activity not only during action observation but also during the preparation of movements (Zimmermann et al. 2018; Monaco et al. 2019). fMRI studies further indicate that EBA activity reflects proprioceptive body‐part representation (Limanowski and Blankenburg 2016; Limanowski and Friston 2020) and contributes to early stages of motor planning (Astafiev et al. 2004). Moreover, joint representations of the to‐be performed motor action and the effector have been decoded from this region during motor planning (Gallivan, McLean, et al. 2013). Together, the IPS and EBA may support an early stage of motor planning in which sensory information is represented in an effector‐specific, motor‐relevant reference frame (i.e., in body‐centred coordinates) (Gallivan and Culham 2015). Coherently, fMRI MVPA studies found information coding in both regions prior to the primary motor cortex (M1)—a region critical for movement execution (e.g., Andrushko et al. 2021), preparation, and planning (e.g., Ariani et al. 2022). This temporal sequence has been interpreted as indicating the IPS's and EBA's contribution in specifying action parameters, such as the intended motor output (Monaco et al. 2020; Rens et al. 2023), before transformation into executable movement plans and subsequent execution.

The process of encoding and transforming intended motor outputs into detailed movement plans likely involves cognitive mechanisms similar to WM transformation, which have been extensively investigated using fMRI decoding techniques (Haynes and Rees 2006; Norman et al. 2006). In particular, the application of time‐resolved MVPA in delayed‐response paradigms has been successful in identifying where and when information is encoded during different phases of WM (Schmidt et al. 2017; Hebart et al. 2018) and motor planning (Gallivan, McLean, Smith, and Culham 2011; Ariani et al. 2018). In WM studies, time‐resolved decoding has indicated transitions in representational content from low‐level sensory features to more abstract representational codes—across visual, tactile, and auditory modalities (Schmidt et al. 2017; Uluç et al. 2018, 2020; Hebart et al. 2018). These studies further indicate that transformations into more abstract format representations occur in higher‐order sensory and multimodal cortices, depending on task demands (Christophel et al. 2017). However, these studies have not yet addressed how this information is translated into a motor plan for execution. Only recently, fMRI MVPA studies have employed time‐resolved approaches to decode action‐related information, as revealed by transformation processes occurring from the selection of an intended action to movement parameters (Caccialupi et al. 2025). In this study, using a delayed grip‐force task, we investigated where and how grip‐force intensities are parametrically encoded in the brain. By applying time‐resolved MVPA, we observed a reformatting process: the intended grip‐force intensity was initially represented in the l‐IPS and then transformed into motor codes of the l‐PMd. This combination of delayed‐response paradigms and time‐resolved MVPA allowed us to track transformation processes over time, revealing that grip‐force intensities are parametrically represented in distributed neural patterns in contralateral brain regions during action selection and movement planning. However, since only one effector was involved in the task (i.e., the right hand), our results did not directly address whether these representations are encoded in an effector‐specific format. Thus, it remains to be systematically tested where and how motor parameters, like graded grip‐force intensities, are represented in effector‐specific brain regions during action selection and movement planning.

The present study thereby investigates where and when effector‐specific grip‐force intensities can be parametrically decoded from fMRI data. We modified our previous paradigm (Caccialupi et al. 2025) and employed a delayed grip‐force task in which grip‐force preparation was cued for either the right or left hand, with the to‐be‐used hand switching halfway through the delay period in 50% of the trials. Using time‐resolved MVPA with support vector regression (SVR), we decoded parametric grip‐force intensities for both hands across two subsequent 6‐s delay periods. Additionally, cross‐decoding analyses were used to test whether grip‐force intensities were represented in an effector‐specific or effector‐independent format. Based on existing literature, we hypothesise that parametric neural representations of grip‐force intensities can be identified in lateralised sections of action‐specific brain regions, such as the contralateral PMds, M1s, and PPCs.

## 2 Materials and Methods

### 2.1 Participants

All participants (*N* = 29) were healthy and right‐handed, as assessed by the Edinburgh Handedness Inventory (EHI; Oldfield 1971). Before the experiment, they provided written informed consent to participate in the study in accordance with protocols approved by the local ethics committee of Freie Universität Berlin (003/2021, Berlin, Germany).

To account for potential biases and confounds, only *N* = 25 participants were included in the analysis (age: 30 ± 6.31, 4 female). Four participants were excluded due to frequent grip‐force application during the delay period or task performance with the wrong hand (i.e., percentage of trials > 33.3% in at least one run and combination of effector cue, force intensity and delay period). To further ensure that the analyses were not confounded by early motor responses, we excluded from the dataset all trials in which any grip‐force was applied during the delay period (cut‐off: mean grip‐force ≥ 0.05 on a scale from 0 to 1).

### 2.2 Experimental Procedure

**FIGURE 1:**
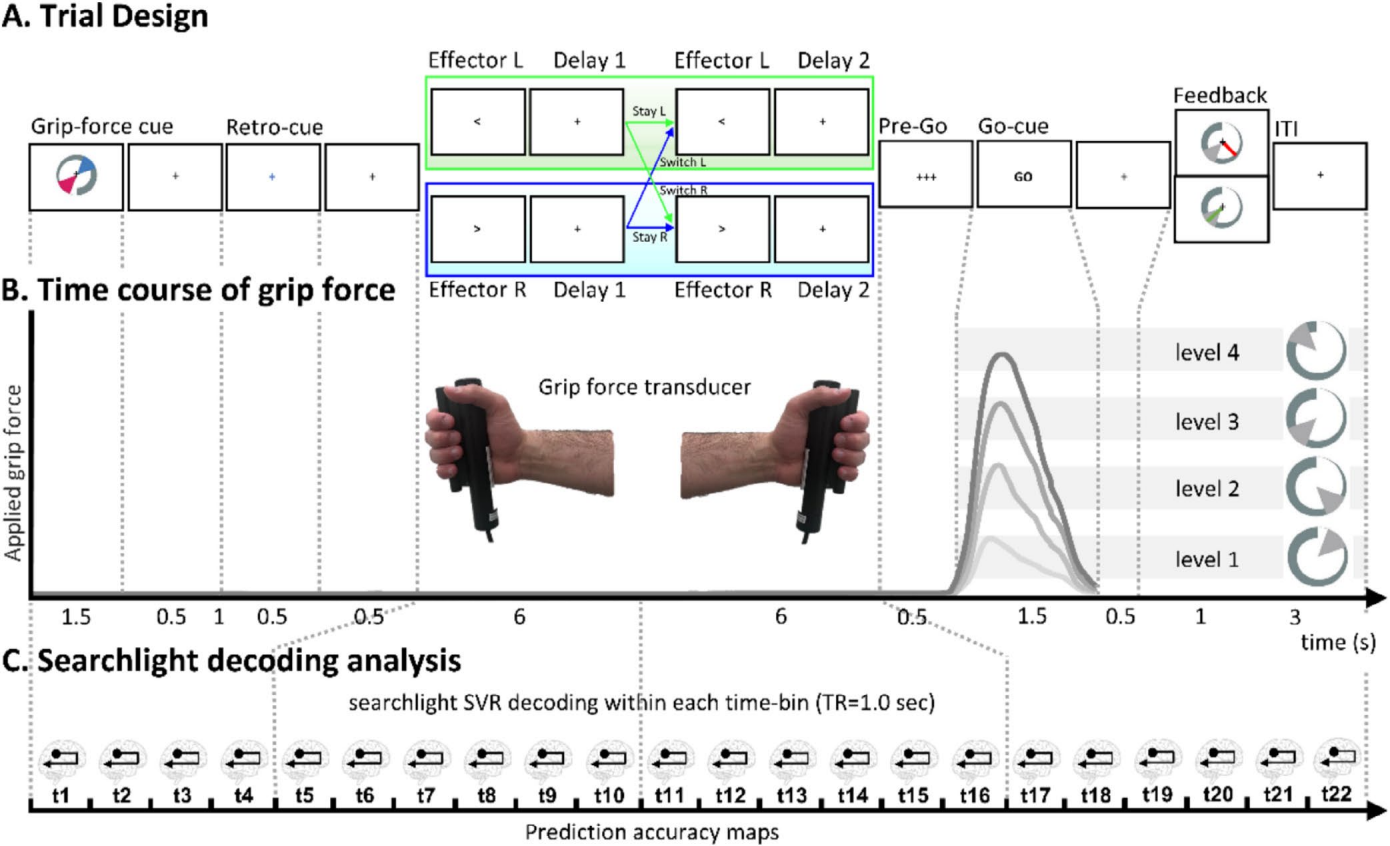
(A) Delayed grip‐force paradigm. Two grip‐force levels were presented on a *Grip‐force cue* (i.e., a grey circular increasing bar with one level represented in cyan and the other in red, as sectors of 70° on the indicator). A *visual Retro‐cue* (i.e., a cyan or red cross displaced in the centre of the screen) indicated which grip‐force had to be anticipated and maintained during two subsequent 5.5 s delay periods. A 0.5 s *Effector cue* (i.e., a left or right arrow at the fixation cross) was presented at the beginning of each delay period. This cue indicated whether the grip‐force should be prepared with the left hand (*Effector L*) or the right hand (*Effector R*). After the second delay, participants prepared and performed the grip force upon the presentation of a *Pre‐Go* (0.5 s) and a *Go‐cue* (1.5 s). Subjects responded with either their right or left cued hand by squeezing a grip‐force device with the cued intensity, and a *Feedback* representing the applied force was provided at 2 s from the *Go‐cue*. (B) Different shades of grey represent time courses of mean grip forces applied during the experimental‐trial, calculated over the trials per condition (i.e., per grip‐force level) and averaged across hands. Light‐grey bars displayed in the background represent grip‐force levels. (C) Representation of the multivoxel pattern analysis (MVPA) with a searchlight and a time‐resolved approach adopted across four time periods of the trial, including: A 4 s *cue period*, 6 s *first delay period*, a 6 s *second delay period*, and a 6 s *motor execution period*, *feedback* and *ITI*.

After participants entered the MRI scanner, the grip‐force transducer was calibrated to participants' maximum grip‐force (i.e., the average of the maximum force applied by the right and left hands). Next, participants were introduced to the four grip‐force levels that served as the target grip‐force intensities in the main experiment. In this training phase, they were familiarized with the grip‐force transducer by performing sixteen trials with real‐time visual feedback of the applied grip‐force. In thirty‐two further trials, they were trained on the experimental task (see below) during the acquisition of a structural MRI scan. Finally, participants performed the delayed grip‐force task (illustrated in Figure 1A) in four runs during fMRI scanning.

### 2.3 Grip‐Force Assessment and Stimuli

Based on the visual presentation of a *grip‐force cue*, a *retro‐cue*, and an *effector‐ cue* participants had to apply a grip force either via the right hand or the left hand on a cylindrical MR‐compatible grip‐force transducer (i.e., a *force fibre optic response pad*, Current Designs, HHSC‐1x1‐GRFC‐V2; illustrated in Figure 1B). Grip‐force intensity was sampled throughout the task to ensure that participants only applied force during the execution period of the trials, allowing the exclusion of trials where participants applied force during other periods of the experimental trials.

**FIGURE 2:**
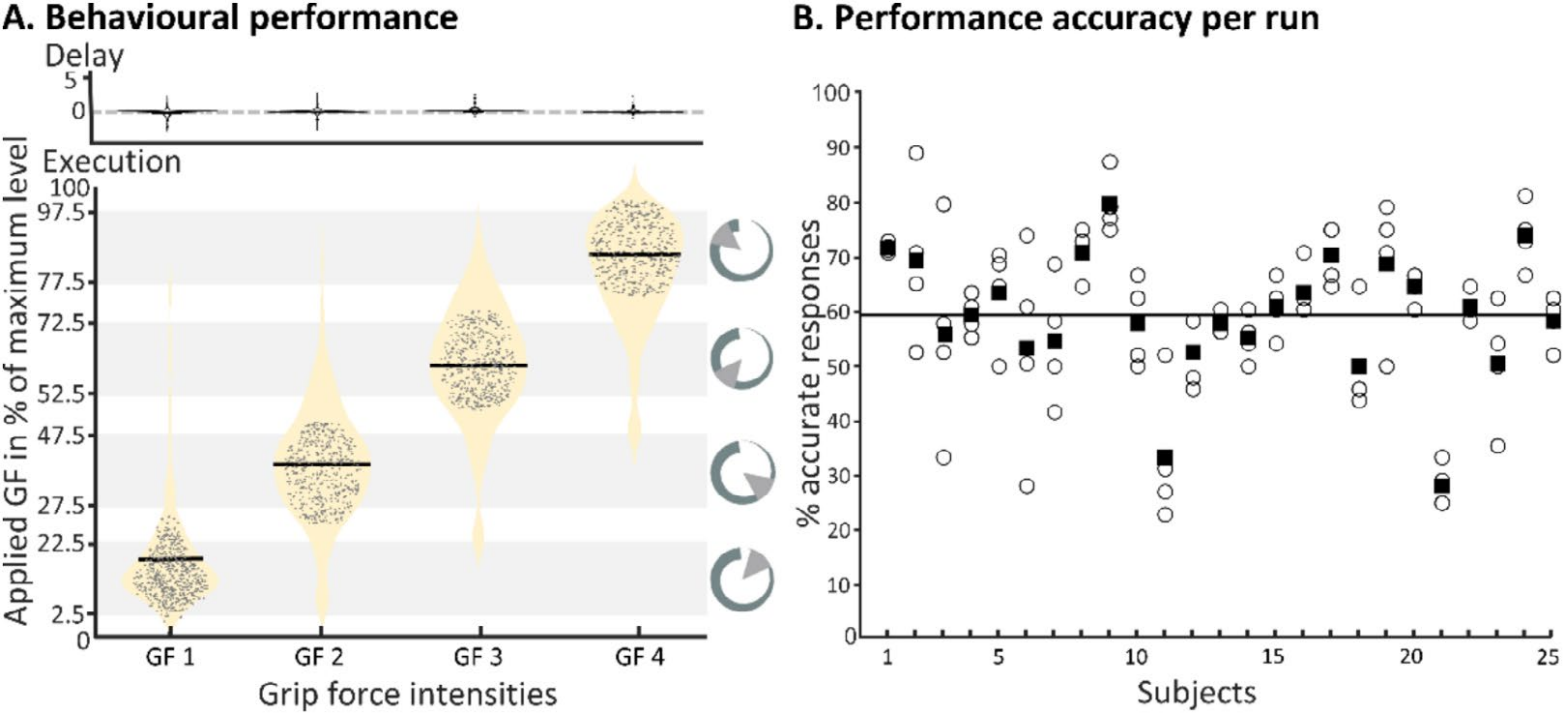
Behavioural assessment. (A) Displays the grip‐forces applied during the delay period (upper display) and the execution period (lower displays). We made sure that only trials were included in the analysis, where participants did not press the grip‐force device during the delay periods (upper panel), and grip‐force was considered accurate when it did not exceed or fall short by more than 4.15% of the target intensity (lower panel). Light grey bars in the background represent the cued grip‐force (GF) levels. For each of the four cued GF levels a violin plot (beige) illustrates the distributions of applied grip‐forces, with accurate trial performances indicated as black dots. (B) Shows that participants performed broadly consistently across four runs of the delayed grip‐force task. Circles represent the performance of accurate grip‐force application in each of the four runs (for each participant); filled squares represent the mean performance across runs. The group's overall mean performance is represented by the black line.

The visual *grip‐force cue* was presented in the centre of the screen, utilizing *Psychtoolbox‐3* (Brainard 1997). The visual cues were composed of a 360° circular display increasing in thickness, corresponding to an increase in grip‐force where the maximum corresponded to 75% of the individual maximum grip‐force. Four grip‐force levels were indicated by the display of a range, defining a sector of 70° of the grip‐force spectrum, with grip‐force level 1: 10°–80° (2.5%–22.5% of maximum grip force level), level 2: 100°–170° (27.5%–47.5%), level 3: 190°–260° (52.5%–72.5%), and level 4: 280°–350°(77.5%–97.5%) (as illustrated in Figure 1B, right side; and Figure 2B). The grip‐force cue was presented for each trial with a random degree of rotation (as illustrated in Figure 1A).

### 2.4 Experimental Task

During fMRI scanning participants performed a delayed grip‐force task. Each trial started with the presentation of a *grip‐force cue*, comprising of two grip‐force levels presented in cyan and red (illustrated in Figure 1A). A *retro‐cue* indicated which of the two grip‐force intensities had to be maintained and executed. The combinations of displayed grip‐force levels were balanced so that all combinations of two different levels were presented equally often. Two 0.5 s *effector cues*, respectively presented at 0 s and 6 s from the beginning of a 12 s *delay period*, indicated which hand to prepare for execution: the right hand in 50% of trials and the left hand in the other 50%; and in 50% of the cases, the hand to be used had to be switched from the initial side to the other side after the second effector cue. Upon the display of a *Go‐cue*, participants applied the anticipated grip‐force with the last cued hand; importantly, in the absence of a grip‐force or effector cue. A *pre‐Go‐cue* was presented for 0.5 s before the response to allow optimal response timing during the 1.5 s response window. Finally, visual *Feedback* was provided by showing on a screen the applied force as a green radian if accurate or as a red radian if inaccurate, that is, outside of the cued grip‐force level (compare Figure 1A). The mean grip‐force during the last 0.75 s of the response window was evaluated to assess accuracy of responses. For the analysis, trials were considered accurate when the applied force was within the target range ±15° (4.15% of maximum grip force).

Four experimental runs comprised 48 experimental trials supplemented with 12 catch trials with shorter delay periods of respective durations: 5.5 s and 2 s, and 2 s and 0 s. The second effector cue was not presented when the duration of the first delay was reduced. Catch‐trials were designed to enhance participants' readiness to prepare and execute the grip‐force task, and thus the continuous maintenance of the force intensity. Catch trials were not included in the analyses. Trial order was fully randomized within a run, where each experimental condition (i.e., the cueing of each of the four to‐be anticipated grip‐force intensities and effector cues) was presented equally often (i.e., 3 times per run).

### 2.5 fMRI Data Acquisition and Preprocessing

fMRI data were acquired in 4 runs of 20 min and 7 s each on a 3 T Siemens Prisma at the Center for Cognitive Neuroscience Berlin (CCNB) of the Freie Universität Berlin. For each run, 1313 functional images were obtained with an EPI sequence (64 channel head coil, 48 slices, interleaved order; TR = 1 s, 2 × 2 × 2 mm voxel size; multiband acquisition with acceleration factor of 3).

Trial onsets were time‐locked to the functional image acquisition to allow a time‐resolved analysis (see below). MRI data processing was performed using SPM12, r7771 (Wellcome Trust Centre for Neuroimaging, Institute for Neurology, University College London). To preserve the spatiotemporal structure of the fMRI, data preprocessing was limited to spatial realignment.

#### 2.5.1 Time‐Resolved Searchlight Decoding of Effector‐Specific Grip‐Force Anticipation

We employed an MVPA searchlight approach to identify brain regions that exhibited multivariate parametric codes of effector‐specific grip‐force anticipation (i.e., distinct brain‐activity codes for each to‐be‐used hand). A finite impulse response (FIR) model was used to obtain run‐wise beta estimates for each 1 s time‐bin of the trials. Twenty two consecutive time‐bins were modelled (as illustrated in Figure 1C), comprising 6 time‐bins throughout each delay period, 4 additional time‐bins before the first delay period and 6 time‐bins from grip‐force execution. High‐pass filtered data (cut‐off 192 s) were included in a first‐level GLM, modelling grip‐force preparation for each combination of intensity and hand in 732 regressors (8 conditions × 22 time‐bins × 4 runs complemented with the 6 realignment parameters).

To identify where and when information about the maintained grip‐force was encoded in the brain, we applied time‐resolved MVPA using SVR (Kahnt et al. 2011), which can be considered a multivariate pendant of parametric coding, a method previously applied in a series of WM decoding experiments (e.g., Christophel et al. 2012; Schmidt et al. 2017; Uluç et al. 2020; Pennock et al. 2021). Using a searchlight approach (*r* = 4 voxel) independently for every time‐bin allows testing for local multivariate representations (i.e., activation patterns of voxels) that code parametric grip‐force anticipations (Caccialupi et al. 2025). Four SVR models were trained to predict grip‐force (i.e., the four grip‐force intensities) based on a multivariate data vector (i.e., multivoxel activation pattern) for each combination of delay period and effector cue (i.e., D1R, D1L, D2R, D2L). To account for differences in the difficulty of grip‐force performance, the distances between the four SVR labels were adjusted using Fechner's law (Fechner, 1860) as previously done (see Caccialupi et al. 2025).

Beta estimates of each condition were first normalized (i.e., z‐scaled) across the samples for each voxel as implemented in the Decoding Toolbox (TDT; Hebart et al. 2015) and forwarded to a four‐fold leave‐one‐run‐out cross validation schema. To make our data comparable to previous reports, we used as measure the prediction accuracy defined as the Fisher's z‐transformed correlation coefficient between the predicted value levels and the actual value levels of the test dataset (in TDT: “zcorr”, see also Kahnt et al. 2011). The centre of the searchlight was moved voxel wise through the brain, and prediction accuracy values were saved as corresponding whole‐brain accuracy maps. In this way, we obtained an accuracy map for every subject and time‐bin, reflecting local activation patterns that code effector‐specific grip‐force intensity in a multivariate way.

Prediction accuracy maps were entered into a second‐level analysis to test for above‐chance decoding in terms of SPM's flexible factorial design implementation of an ANOVA. We used a t‐contrast to test for above chance decoding across different time‐bins. All results are reported at a threshold of *p* < 0.05 family‐ wise error (FWE) corrected at the voxel level.

#### 2.5.2 Cross‐Decoding Analyses: Cross‐Hand Cross‐Decoding Versus Switch/No‐Switch Cross‐Decoding

We conducted two types of cross‐decoding analyses. First, Cross‐Hand cross‐decoding analyses (i.e., X‐Dec: Cross Hand) were performed to test whether parametric representations of grip‐force intensity are found in an effector‐independent format (i.e., information about left and right hands would be retained as the same brain activation pattern). In contrast, if information is retained in an effector‐specific format, it should not be possible to cross‐classify, as codes for left‐hand preparation should be stored in different regions than codes for right‐hand preparation. The logic underlying the use of SVR in this type of cross‐decoding is the same as in the main analysis, with the only difference being that in X‐Dec: Cross Hand, training and testing of the SVR model were conducted on beta estimates corresponding to different cued hands. The SVR model was first trained on beta estimates reflecting preparation with the right hand and tested on the left hand, then vice versa.

Secondly, we performed Switch/no‐Switch cross‐decoding analyses (i.e., X‐Dec: Switch/no‐Switch), in which training and testing were carried out on beta estimates corresponding to the same hand. This analysis tested whether the decoding in effector‐specific regions in the second delay period differs if it is preceded by a switch of sides or not. For example, preparing with the left hand in the second delay period after a prior left‐hand cue in the first delay period (i.e., a no‐switch condition) may elicit different activation patterns as compared to the situation where a prior right‐hand cue for the first delay period was provided. This analysis solidifies the effector specificity of the findings in the main analysis if the effector‐specific codes in the second delay period can be found even in the cross‐decoding. Any cross‐decoding analysis was based on a similar FIR model as in the main decoding analysis. However, here the first‐level GLM modelled each combination of effector cues (across delay periods) and grip‐force intensities, resulting in a first‐level GLM with 1432 regressors (16 conditions × 22 time‐bins × 4 runs, complemented by the 6 realignment parameters). The same four‐fold leave‐one‐run‐out cross‐validation schema employed in the main decoding was used, generating 22 prediction accuracy maps for each sequence combination of effector cues (i.e., RR × RL, RR × LR, RR × LL, RL × LR, RL × LL, LR × LL). For each SVR model, the distances between the four SVR labels were adjusted, as in the main analysis, using Fechner‐corrected labels.

After normalization and smoothing, prediction accuracy maps were entered into second‐level analyses to test for above‐chance decoding using SPM's flexible factorial design implementation of an ANOVA. The first analysis included maps for sequences with different effector‐cue combinations during the first delay (RR × LR, RR × LL, RL × LR, RL × LL), while the second focused on the later delay (RR × RL, RR × LL, RL × LR, LR × LL). Each second‐level design comprised 4 factors and either 10 or 12 levels for time bins (t1–t10 and t11–t22, which respectively model the initial phase of the trial, up to the end of the first delay, and the second part). T‐contrasts were used to assess above‐chance decoding on maps of the second half of each period, as done in the main analysis. A conjunction analysis over t‐contrasts was tested against a conjunction null hypothesis (Nichols et al. 2005) to identify regions representing effector‐independent information. All results are reported at *p* < 0.05 FWE‐corrected at the voxel level.

#### 2.5.3 Control Analyses: Label Permutation Control Analysis

To control whether prediction accuracies of the main analysis were based on the parametric coding of the grip‐force intensity, we performed a label‐permutation test. Here, the labels of the conditions entered into SVR‐models were permuted. As previously applied (see Schmidt et al. 2017; Uluç et al. 2020; Caccialupi et al. 2025), higher distance of the labelling from the original order should result in reduced decoding accuracies (getting to zero for the highest distance that is, completely unordered labelling). For all possible permutations, the distance from the rank order was calculated as the sum of the absolute difference of adjacent ranks (e.g., the linear order of grip‐force level 1, 2, 3, 4 has a distance of ranks of sum (|1–2| + |2–3| + |3–4|) = 3 and the permuted labelling 2, 1, 3, 4 corresponds to the sum (|2–1| + |1–3| + |3–4|) = 4, resulting in a difference of 1 from the linear order). Thereby, the permutation analysis congregated permutations into four classes of distances from the linear order. For all permutations of labels, the same SVR whole‐brain searchlight analyses as in the main decoding were carried out.

To test for above‐chance decoding on the maximally permuted label, prediction accuracy maps were entered into the same second‐level analysis used in the main decoding. Additionally, to assess whether prediction accuracy increased parametrically with decreasing distance from the linear order of labels, we computed four second‐level flexible factorial designs. These included one factor for label type (Ordered, Distance 1, Distance 2, Distance 3, and Distance 4) and a second factor for time bins (either t1–t10 or t11–t22). T‐contrasts were used to test for above‐chance decoding within each time period. All results are reported at *p* < 0.05, FWE‐corrected at the voxel level.

#### 2.5.4 Control Analyses: FIR‐Model Based Univariate Analysis

We investigated parametric increases in brain activity during the two delay periods by conducting a univariate control analysis. For consistency, this analysis was based on the same FIR model used in the main decoding analysis, with the only difference being that functional images were normalized and smoothed (using SPM's default 8 mm FWHM kernel) before entering the first‐level model. For each subject, 176 contrast images were computed (8 combinations of effector cues and force intensities × 22 time bins) by averaging beta estimates across runs and weighting them using “Fechner‐corrected” labels (i.e., −2.40, −1.48, 0.45, and 3.42).

These contrast images were then entered into four independent second‐level flexible factorial designs, following the same logic as the main analysis. Each design included a single factor (corresponding to right‐ or left‐hand preparation) and either 10 or 12 levels for the time bins (t1–t10 and t11–t22, respectively). Finally, t‐contrasts were computed for different time periods of the experimental trials (see above). All results were reported at *p* < 0.05, FWE‐corrected at the voxel level.

## 3 Results

### 3.1 Behavioural Performance

The participants included in the main analysis (*N* = 25) responded with accurate application of grip‐force (i.e., they were in the target range ±15°) in 59.8% ± 15% (mean ± SD) of the trials. To test for potential differences in performance across hands and grip‐force intensities, we conducted a 2 × 4 repeated‐measures ANOVA. This analysis revealed no significant main effect of hand (df = 1, *F* = 3.11, *p* < 0.095, *η* ^2^ = 0.019), but a significant main effect of force intensity (df = 1, *F* = 421, *p* < 0.001, *η* ^2^ = 0.93), as well as a significant hand × intensity interaction (df = 3, *F* = 7.34, *p* < 0.012, *η* ^2^ = 0.06). Post hoc *t*‐tests revealed no significant differences in performance between hands at any force intensity. In contrast, for both hands, trials involving the lowest grip‐force intensity were slightly easier than those requiring higher intensities. Bonferroni‐corrected *p*‐values showed that mean performance accuracy with the left hand was significantly higher for intensity 1 compared to intensity 2 (*M*_diff_ = 22%, *p* = 0.022), 3 (*M*_diff_ = 32%, *p* < 0.001), and 4 (*M*_diff_ = 16%, *p* < 0.001). Similarly, with the right hand, accuracy for intensity 1 was significantly higher than for intensity 2 (*M*_diff_ = 6%, *p* = 0.022) and 3 (*M*_diff_ = 16%, *p* < 0.001). These findings are consistent with those of a previous study by Caccialupi et al. (2025). Accordingly, we applied an adjustment based on Fechner's Law to model the subjectively perceived differences in grip‐force intensity for the neuroimaging analyses (see Caccialupi et al. 2025). As in this study, participants' robust performance accuracy (~60%, well above the 25% chance level) indicates reliable grip‐force control. Violin plots in Figure 2A display the distribution of responses (i.e., accurate and non‐accurate performances) in terms of applied force on the grip‐force device for the four grip‐force intensities (averaged between hands, due to not significant differences). We carefully ensured that the included participants did not apply force during the delay period (see Figure 2A, upper display).

To assess potential training or fatigue effects across the four runs, we conducted a 1 × 4 repeated‐measures ANOVA (averaging performance accuracies across hands), which did not reveal differences (df = 3, F = 2.73, *p* = 0.0491, *η* ^2^ = 0.0178). Performance accuracy per participant and run is illustrated in Figure 2B.

### 3.2 Multivariate Mapping of Regions That Code Effector‐Specific Grip‐Force Anticipation

**FIGURE 3:**
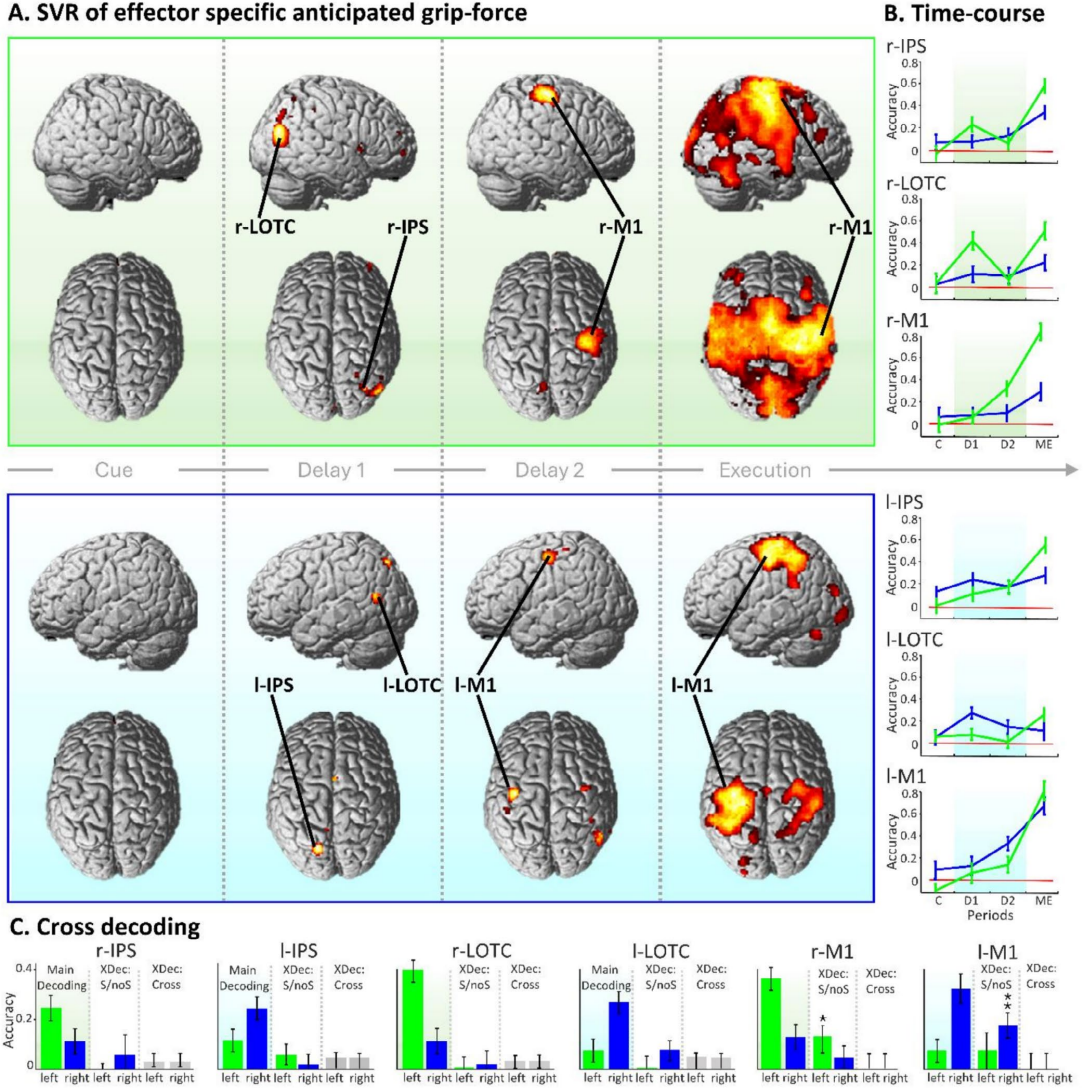
Results of time resolved support vector regression analyses (A) Displays brain regions that parametrically code grip‐force intensities during the cue period (C), first delay period (D1), second delay period (D2) and motor execution period (ME). The upper, green panel indicates preparation with the left hand; the lower, blue panel, preparation with the right hand. Brain regions with above‐chance prediction accuracy are revealed by t‐contrasts testing the respective periods against zero, at *p* < 0.05, FWE‐corrected. As expected, no brain region exhibited above‐chance decoding during C (first column). In D1, two lateralized clusters were revealed in the IPS and LOTC, on contralateral hemispheres respectively. In D2, we found contralateral M1 (third column), indicating transformation of neural codes prior to execution. Finally, t‐contrasts on the ME revealed contralateral and ipsilateral clusters in the M1s and S1s (fourth column). (B) Time‐courses of prediction accuracy for left‐ and right‐hand preparation (represented in green and blue) were extracted from peak voxels identified in the main analysis, in contralateral r‐IPS [*x* = 24, *y* = −58, *z* = 58], l‐IPS [*x* = 42, *y* = −72, *z* = 18], r‐LOTC [*x* = −16, *y* = −74, *z* = 48], l‐LOTC [*x* = −40, *y* = −64, *z* = 14], r‐M1 [*x* = 36, *y* = −20, *z* = 62] and l‐M1 [*x* = −40, *y* = −16, *z* = 54]. Please note that because time‐courses were extracted from the most significant voxels they are only displayed for descriptive purpose. No further statistical testing was conducted to avoid circular conclusions. (C) Displays prediction accuracies after left‐hand (green) and right‐hand (blue) effector‐cue presentations. To assess effector‐specific and effector‐independent coding in the IPS, LOTC, and M1 regions, we conducted two cross‐decoding analyses: *X‐Dec: Cross Hand* (Cross) and *X‐Dec: Switch/no‐Switch* (S/noS). Prediction accuracies are shown for the main decoding (left panel, coloured background), *X‐Dec: Switch/no‐Switch* (middle panel), and *X‐Dec: Cross Hand* (right panel, grey bars). To avoid circularity in statistical testing, prediction accuracies were extracted from peak voxels identified in the main decoding analysis (Figure 3B, Table 1): IPS and LOTC during the first delay period, and M1 during the second. Please note that because these prediction accuracies were extracted from the most significant voxels, the bar plots for the main decoding are shown for descriptive purposes only, to illustrate relative effect sizes of the main decoding and cross‐decoding analyses. Significance testing is not reported here to avoid circularity, which could lead to overestimation of significance values. One‐sample t‐tests showed that above‐chance decoding—Bonferroni‐corrected *p*‐values: *p < 0.05, **p < 0.01—was restricted to contralateral regions. In the *X‐Dec: Switch/no‐Switch* analyses, above‐chance decoding was consistently observed in contralateral regions involved in preparing the target hand during the second delay period. In contrast, the absence of significant results during the first delay period (where information was cross‐decoded between hands), and in the *X‐Dec: Cross Hand* analyses during both delays, suggests that grip‐force preparation is not represented in similar activation patterns across hands. This supports the view that effector‐independent coding is unlikely in the IPS and LOTC during the first delay, and in M1 during the second delay.

To identify brain regions encoding parametric grip‐force anticipation in two delay periods, we conducted a time‐resolved whole‐brain SVR analysis. Within a second‐level ANOVA design, we computed t‐contrasts across prediction accuracy maps for the three trial periods of interest: the cue period (i.e., t3–t4), the first delay period (t8–t10), and the second delay period (t14–t16). T‐contrasts were computed on the second half of each period to account for temporal correlations of the BOLD‐signal; all results are reported at *p* < 0.05 FWE‐corrected. During the *cue period*, as expectable, no brain region exhibited above‐chance decoding, even when assessed at p < 0.001 uncorrected, demonstrating the specificity of the other findings. During the first delay period, two lateralized clusters comprising contralateral intraparietal sulcus (IPS) and extrastriate body area (EBA) were found in the right and left hemispheres during preparation with the left and right hands, respectively (see Figure 3A, Table 1). During the second delay period, we found strong above‐chance decoding in the contralateral primary motor cortices (M1), corresponding to left‐ and right‐hand preparation conditions. The lower decoding accuracy observed in the ipsilateral M1s may be attributed to their known specialization in modulating and refining movement plans (Ghacibeh et al. 2007), or to the inhibition of the non‐cued hand (Pool et al. 2014; Tzourio‐Mazoyer et al. 2015). Finally, a control t‐contrast on the motor execution period (i.e., t20‐t22) revealed expected large bilateral M1s and primary somatosensory cortices (S1) clusters, with highest significance in the respective contralateral hemispheres (Figure 3A, fourth column, and Table 1). Analogous results were obtained when group‐level statistics were performed on prediction accuracy maps derived from accurate trials only (computed following the same procedure as the main decoding but on a subset of participants; see Figure S3A in the Supporting Information), suggesting that variability in performance accuracy is unlikely to have influenced the main SVR results.

**TABLE 1:** Regions that exhibit above‐chance prediction accuracy across the cue period, first and second delay periods, and motor execution period, revealed by a t‐contrast displayed at *p* < 0.05, FWE corrected.

Cluster size	Anatomical region	Peak MNI coordinates			
		*x*	*y*	*z*	z‐score
First delay period, left hand					
734	Right LOTC	44	−72	18	6.62
12	Right IPS	24	−58	58	4.77
52	Right FO	38	10	12	4.69
17	Right MFG	38	58	0	4.61
6		38	54	20	4.59
First delay period, right hand					
162	Left IPS	−16	−74	48	5.64
144	Left LOTC	−40	−64	14	5.27
21	SMA	2	2	54	4.74
12	Left PCu	−6	−52	50	4.72
Second delay period, left hand					
1152	Right M1	36	−20	62	6.74
62	Left IPS	−8	−68	52	4.82
6	SMA	8	−8	52	4.53
1	Left M1	−58	−14	44	4.50
Second delay period, right hand					
185	Left M1	−40	−16	54	5.68
207	Right AngG	50	−58	28	5.23
38	Right M1	34	−10	62	4.75
18	Left S1	−46	−34	64	4.65
Motor execution period, left hand					
70,631	Right M1	30	−22	56	11.93
583		−38	48	8	5.59
383		38	28	40	5.31
Motor execution period, right hand					
5961	Left M1	−32	−26	54	8.17
	Left S1	−40	−36	54	7.45
2437	Right M1	50	−26	52	6.28
	Right S1	34	−36	58	5.66

To plot the temporal evolution of effector and grip‐force specific decoding accuracy within brain regions recruited during the two delay periods, we extracted the time‐course of prediction accuracies from the peak voxels of the identified clusters in the IPS, LOTCs, and M1s (see Table 1 for MNI coordinates). Each time‐course represents four prediction accuracy values obtained by averaging prediction accuracy values of twenty‐two time‐bins in correspondence of those tested in the four time‐periods. Time‐courses of mean prediction accuracies within the four time‐periods of the experimental trials are displayed in Figure 3B (similar to Caccialupi et al. 2025). As expected, time‐courses systematically showed above‐chance grip‐force intensity anticipation in contralateral brain regions. Consistent with results of group‐level analysis, time‐courses in the LOTCs and IPS displayed an early peak during the first delay period, preceding those of contralateral M1s, which reached maximum accuracy during the second delay. These expected kinetics underlie changes in accuracy within effector‐specific regions (i.e., regions showing systematic lateralization for movement preparation with the right and left hands), which indicates the high specificity of the reported effects and the likely effector‐specific encoding of anticipated grip‐force intensities. Together with group‐level statistical testing, these results indicate regions where information is contained in a parametric‐ and effector‐specific format. Additionally, it illustrates the likely contribution of secondary sensory regions to effector‐specific grip‐force intensity anticipation prior to M1s. Each reported region showed a substantial increase in prediction accuracy during motor execution.

### 3.3 Testing for Effector‐Specific Lateralization Effects by Cross‐Decoding

To assess whether grip‐force intensities are represented by similar brain activation patterns during preparation for both the right and left hands, we conducted whole‐brain cross‐decoding analyses. Force intensities were decoded using an SVR model trained on beta estimates reflecting motor preparation with one hand and tested on the other (i.e., trained on the right hand and tested on the left, and vice versa). Within a second‐level ANOVA design, t‐contrasts were computed on prediction accuracy maps for the same time periods tested in the main decoding analysis. However, a conjunction analysis (tested against a conjunction null hypothesis, to inspect above‐chance decoding across all effector‐cue sequence combinations involving different hands within a target delay period) revealed no brain regions with above‐chance decoding, even at an uncorrected threshold of *p* < 0.001 (Figure 3C) or *p* = 0.01. This result makes it unlikely that the above‐chance decoding found in the main analysis reflects preparation of ipsilateral hand movements, as that would require cross‐hemispheric information coding for each hand and, plausibly, above‐chance cross‐decoding. Additionally, it undermines the hypothesis that anticipated grip‐force intensities were parametrically represented in an effector‐independent format.

To further corroborate the results of the main and *X‐Dec: Cross Hand* analyses, we conducted *X‐Dec: Switch/no‐Switch* analyses to test whether lateralization effects observed during the second delay could be replicated using an alternative FIR model, and whether similar activation patterns for the two hands were present during the first delay. To this end, in the first‐level GLM, trials were modelled using independent regressors based on whether the cued‐hand switched or did not switch during the second delay. Following whole‐brain cross‐decoding, prediction accuracy values were extracted from the peak voxels identified in the main analysis (Table 1) and are displayed in Figure 3C (middle bars). To assess above‐chance decoding in the left and right IPS and LOTC during the first delay period (when training and testing were conducted on betas corresponding to different hands), and for switch versus no‐switch conditions during the second delay in the M1s (for each hand independently), we performed four one‐sample *t*‐tests across participants (2 decoding types × 2 hands) for each peak voxel. Bonferroni‐corrected *p*‐values (corrected for four tests) confirmed that above‐chance decoding was restricted to contralateral M1s during the second delay, with significant effects in the r‐M1 (left hand, *p* = 0.037) and l‐M1 (right hand, *p* = 0.006), as shown in Figure 3C (black asterisks). The absence of above‐chance decoding during the first delay—when training and testing were conducted on beta estimates corresponding to two different hands—is consistent with the results of both the main decoding and *X‐Dec: Cross Hand* analyses and further supports the lateralized nature of grip‐force encoding. As expected, decoding performance was slightly lower compared to the main analysis, likely due to the smaller number of trials modelled in the first‐level GLM (reduced from six to three per condition). Nonetheless, the consistent above‐chance prediction accuracy in contralateral M1s reinforces the robustness of the main decoding results.

Taken together, the *X‐Dec: Cross Hand* and *X‐Dec: Switch/no‐Switch* analyses provide converging evidence for effector‐specific coding of parametric information in the IPS, LOTCs, and M1s.

### 3.4 Control Analyses: Label Permutation Test

To corroborate specificity of the main analysis, we conducted label permutation testing and two second‐level analyses. First, we tested above‐chance decoding for the maximally permuted labels in terms of SPM's flexible factorial design implementation of an ANOVA. Convincingly, when t‐contrasts are computed on the first and second delay periods (as in the main analysis), we found no significant clusters throughout the whole brain with identical *p* < 0.05, FWE correction. The same result was observed when inspected at *p* < 0.001 uncorrected. For illustrative purposes, we display the time‐courses of the label‐permutation tests with increasing dissimilarity to the original order for the peak voxels of the main analysis (see Figure S1B in the Supporting Information). As expected, the time‐course of the completely unordered labelling do not show above chance prediction‐accuracies throughout all phases of the experimental trials.

As a second control analysis, we entered prediction accuracy maps resulting from permutation labelling into four second‐level flexible factorial designs (as in the main analysis). Each design included one factor for label type (i.e., Ordered, Distance 1, Distance 2, Distance 3, and Distance 4), and a second factor for time bins (either t1–t10 or t11–t22). We computed parametric contrasts over maps corresponding to the same time bins tested in the main analysis to statistically assess whether prediction accuracies in similar brain regions showed a parametric decrease with increasing dissimilarity of labels from the original intensity order. When assessed at *p* < 0.05, FWE‐corrected, the results corroborated the main findings, showing highly similar clusters across all four time periods (see Figure S1A and Table S1). This indicates that the above‐chance prediction accuracies observed in the main analysis were likely driven by the parametric encoding of grip‐force intensity.

### 3.5 Control Analyses: FIR‐Model Based Univariate Analysis

To test for parametrically modulated activation strength during the two delay periods and motor execution, we performed a univariate analysis based on a first level FIR model. Within a second‐level ANOVA design, t‐contrasts computed over parametrically weighted contrast images reflecting periods preceding motor execution revealed no significant parametric modulation (at *p* < 0.05, FWE‐corrected). During motor execution, only the contralateral r‐M1 and l‐M1 exhibited parametric activity modulation (see Figure S2A and Table S2). These results make it rather unlikely that parametric effects found in the main MVPA analyses were primarily driven by univariate effects.

## 4 Discussion

In this fMRI study, we used a delayed grip‐force task and time‐resolved MVPA to identify brain regions in which grip‐force intensities are parametrically encoded. In particular, we tested whether parametric codes of grip‐force intensities were represented in brain regions that map the to‐be‐used body part (i.e., right or left hand) and how these representations were transformed from a cue period, across two delay periods, up to motor execution.

As expected, information about the intended grip‐force intensity could only be decoded starting from the first delay period (4.5 s after the retro‐cue presentation), which is consistent with the dynamics of the BOLD response, expected to peak around 3–5 s after event onset. In the first delay, we found above‐chance decoding in the contralateral IPS (r‐IPS and l‐IPS) and LOTCs (r‐LOTC and l‐LOTC). Meanwhile, the second delay period revealed contralateral M1s—specifically, the r‐M1 during preparation with the left hand and the l‐M1 during preparation with the right. The systematic lateralization of above‐chance decoding in brain regions contralateral to the to‐be‐used hand indicates that anticipated grip‐force intensities are encoded in effector‐specific areas. Our main analyses further show that information about the anticipated grip‐force intensities is found in different brain regions during the first delay period, as compared to the second delay and motor execution. This likely indicates different coding for the early selection of a motor plan as compared to the actual preparation of the to‐be‐performed grip‐force intensity and execution later in the task. These findings are corroborated by two cross decoding analyses. First, we showed in a searchlight analysis that it is not possible to cross‐decode effector‐specific codes when the SVR model is trained and tested on betas reflecting the preparation of graded grip‐force intensities with different hands (*X‐Dec: Cross Hand*). Secondly, we showed that motor preparation during the second delay leads to the same motor codes, regardless of whether participants directly prepared with one hand or switched to that hand halfway through the trial (*X‐Dec: Switch/No‐Switch*). Taken together, these findings provide strong evidence for the effector specificity of parametric codes found in different brain regions between the first and second delay periods. Moreover, they support the notion of re‐coding over time, transitioning from more abstract (yet action‐specific) codes and effector selection in the IPS and LOTCs to more detailed movement plans and motor preparations in the M1s.

Our results align well with a two‐stage framework of action planning (Boettcher et al. 2021), according to which, after action selection, information about the goal of a motor action is transformed into detailed motor codes. They further support predictions from ideomotor theories, which postulate a contribution of secondary sensory regions to action selection. Specifically, based on our findings, the contralateral IPS and LOTCs likely encode grip‐force information in an effector‐specific format and contribute to the early stages of motor planning (Astafiev et al. 2004; Kühn et al. 2011). As movement planning progresses, the M1s are likely involved in preparing the intended force intensity prior to execution (Gale et al. 2021; Ariani et al. 2022). Within the motor codes of the M1, specific motor parameters—such as the muscles to be used for grip‐force execution—may be represented and maintained in preparation for movement execution (Mizuguchi et al. 2013; Caccialupi et al. 2025).

In the following, we will discuss our findings in the temporal sequence from the cue period to the second delay period.

### 4.1 The Cue Period

No above‐chance decoding was found during the cue period, nor in any primary visual area at any trial phase, even at a liberal threshold of *p* < 0.001 uncorrected. These results indicate that parametric decoding analyses were not influenced by low‐level sensory features of the visual cues. These include the colour‐coding and orientation of the grip‐force indicator—experimentally rendered orthogonal to force intensity levels—and the spatial orientation of the effector indicators. These results support the specificity of the parametric decoding analyses, confirming that above‐chance decoding reflects motor properties of the intended grip‐force rather than perceptual confounds.

### 4.2 The First Delay Period: Action Selection in the Intraparietal Sulcus and the Lateral Occipitotemporal Cortex

During the first delay period, our main decoding analyses revealed two lateralized clusters comprising contralateral IPS and LOTCs, consistently found in the right hemisphere after left‐hand effector‐cue presentation and in the left hemisphere after right‐hand cue presentation.

After right‐hand effector‐cue presentation, the most pronounced cluster was observed in the l‐IPS; vice versa, after left‐hand cue, the corresponding cluster was found in the right hemisphere. Previous fMRI and TMS studies have consistently shown that the contralateral IPS is part of a parieto‐frontal network involved in the planning of grasping actions, even when hand pre‐shaping is not required (Culham et al. 2003; Johnson‐Frey et al. 2005; Tunik et al. 2005; Davare et al. 2009; Gallivan, McLean, Smith, and Culham 2011, Gallivan, McLean, Valyear, et al. 2011, Gallivan, Chapman, et al. 2013, Gallivan, McLean, et al. 2013; Ariani et al. 2018; Ruiz et al. 2024; Gallivan and Wood 2009; Cavina‐Pratesi et al. 2010; Caccialupi et al. 2025). More recent fMRI MVPA studies have found anticipatory brain activity in the IPS and decoded movement properties, such as the grip type (Gallivan, McLean, Smith, and Culham 2011; Gallivan, Chapman, et al. 2013; Gallivan, McLean, et al. 2013; Ariani et al. 2018, 2015; Gallivan, McLean, Valyear, et al. 2011) and the grip‐force (Caccialupi et al. 2025) during a period preceding the execution of a movement. Interestingly, time‐resolved decoding analyses have also found faster increases of prediction accuracy in the IPS, as compared to primary and secondary motor cortices (Gallivan, McLean, et al. 2013; Ariani et al. 2018; Caccialupi et al. 2025). These findings implicate the IPS in the earliest stages of motor planning—such as the encoding of a motor outcome—which might even precede motor preparation (Churchland et al. 2010; Shenoy et al. 2013; Gallivan and Culham 2015; Wong and Haith 2017; Ariani et al. 2018, 2022; Vyas et al. 2020; Ruiz et al. 2024). While information about the to‐be performed action has been decoded in this region regardless of the to‐be used body part (Gallivan et al. 2015), the more posterior section of the IPS (pIPS) has been found to majorly support effector‐specific representation during motor planning (Ariani et al. 2015). On the one hand, above‐chance decoding in the contralateral pIPS systematically lateralized according to the prepared effector (e.g., left vs. right hand; Heed et al. 2016). On the other hand, the contralateral IPS has been found to encode information specific to the intended effector: while decoding is successful for the target hand, cross‐decoding between hand movements and saccades resulted in below‐chance decoding (Gallivan, Chapman, et al. 2013). This functional specialization and consistent lateralization indicate that the contralateral pIPS likely contributes to representing motor actions in an effector‐specific code (Gallivan and Culham 2015).

The role of the posterior pIPS has specifically been discussed as a contribution to the transformation of intended motor outcomes (e.g., reaching to a target position with a specific grip type or force) into specific movement plans (Gallivan, McLean, Smith, and Culham 2011; Gallivan, McLean, Valyear, et al. 2011; Gallivan, Chapman, et al. 2013; Gallivan, McLean, et al. 2013; Barany et al. 2014; Gallivan and Culham 2015). This transformation is likely guided by top‐down attentional mechanisms that are critical for activating and maintaining motor codes in the M1 and selecting to‐be‐executed movements (Calton et al. 2002; Szczepanski et al. 2010; Chapman et al. 2011; Gallivan, McLean, Valyear, et al. 2011; Caccialupi et al. 2025). According to this framework, the pIPS may act as a hub for information processing, where the intended action outcome is represented in an effector‐specific format (i.e., in body‐centred coordinates; Gallivan and Culham 2015) and then transformed into detailed movement plans in downstream motor areas such as the M1 (Ariani et al. 2018, 2022). These findings align with previous fMRI studies reporting the involvement of the contralateral p‐IPS in the anticipatory scaling of the grip force (Van Nuenen et al. 2012), even before the primary and secondary motor cortices are recruited for movement preparation (Caccialupi et al. 2025). Our results further support this view, suggesting that the p‐IPS plays a role in encoding the intended grip‐force intensity during the earliest stages of motor planning—at a time point in which the movement is not yet prepared, but the outcome is represented in an effector‐specific format. This early involvement of the p‐IPS likely aids in the anticipation and maintenance of motor parameters within the contralateral M1, by mapping the motor outcome in a body‐centred reference frame and ultimately translating the intended grip‐force intensity into a specific movement plan (Gallivan, McLean, Smith, and Culham 2011; Gallivan, McLean, Valyear, et al. 2011; Gallivan, Chapman, et al. 2013; Gallivan, McLean, et al. 2013; Gallivan and Culham 2015; Caccialupi et al. 2025).

Beneath the pIPS, we found significant activation patterns in the contralateral LOTCs that reflect to‐be prepared grip‐force intensity during the first delay period. Coherently, fMRI studies have shown that the contralateral LOTC is implicated in motor planning, even during the preparation of unseen motor movements (Lingnau and Downing 2015). In particular, fMRI MVPA studies have shown that activation patterns located in the LOTC might reflect many alternative features of forthcoming hand actions, for example, the motor movement to‐be performed (e.g., reaching in different directions), the action goal (reach to the right vs. to the left) or the effector identity (use the right vs. left hand) (Gallivan, McLean, et al. 2013, 2018). Interestingly, sub‐sections of the LOTC have also been found to encode information about the anticipated perceptual outcomes of the planned motor movement (Lingnau and Downing 2015). To systematically test the specific contribution of LOTC subregions to hand action planning, Gallivan, Chapman, et al. (2013) performed ROI‐based MVPA during the delay periods preceding movement execution. They found that ventral temporal regions, including the FFA, fusiform body area (FBA), and parahippocampal place area (PPA), were involved in encoding motor goals and movement features regardless of the effector. In contrast, joint representations of the to‐be‐performed movements and the effector could be decoded only from lateral occipital regions, such as the lateral occipital cortex (LO) and the EBA. Differently, in our study the activation patterns which reflect effector‐ and force‐specific information have been tested without any a priori assumptions about the regions which might be involved in the task performance. This choice is reflected by our adoption of a searchlight approach rather than a ROI SVR, and by the absence of a dedicated functional localizer for LO or EBA. As a result, we cannot conclusively infer their involvement in our delayed grip‐force task. We chose this approach because, to our knowledge, no previous evidence directly indicates which brain regions underlie the encoding of effector‐ and force‐related information during action selection or motor preparation. Thus, in our study, the involvement of body‐sensitive secondary sensory regions of the LOTC emerged as an intriguing, though not a priori predicted, finding that is nevertheless coherent with previous results (e.g., Gallivan, McLean, et al. 2013). Still, to have found significant patterns in the contralateral LOTC (during the first delay of our grip‐force task) which do not extend to the LO but reflect both the to‐be performed action and the effector, renders more likely the hypothesis that the above‐chance decoding found in the LOCT reflects the implication of the EBA.

The EBA has been found to represent action goals during motor planning (Zimmermann et al. 2016) and encodes body‐part specific information during delay periods (Gallivan, Chapman, et al. 2013). While the EBA activation has been traditionally associated with the visual perception and recognition of non‐facial body parts (Downing et al. 2001), such as hands or feet (Urgesi et al. 2004), it consistently exhibits lateralized activation patterns during the preparation of hand movements (Astafiev et al. 2004; Peelen and Downing 2005; Orlov et al. 2010; Zimmermann et al. 2011, Zimmermann et al. 2016, Zimmermann et al. 2018; Monaco et al. 2019). fMRI studies further indicate that activation patterns in the EBA reflect proprioceptive body‐part representation (Limanowski and Blankenburg 2016; Limanowski and Friston 2019) and might encode the sensory consequences of manual actions based on prior experience (Kühn et al. 2011). Accordingly, the selection of manual actions during motor planning should be mediated by the EBA (but not FFA), as indicated by fMRI findings showing that hand movement preparation selectively recruited hand‐related M1 and EBA sub‐regions (Kühn et al. 2011). These findings align with our results and strengthen the hypothesis that a given sub‐section of the LOTCs, that is, the EBA might play a crucial role in representing the expected sensory consequences of planned actions. Consistent with the ideomotor theory, this suggests that, to initiate a motor action aimed at a specific goal, participants may first anticipate the sensory consequences associated with the action (Elsner and Hommel 2001). This sensory‐based imagery may pre‐activate neural patterns within the EBA, which in turn recruit motor networks previously generating the represented effects. For example, preparing to execute a grip‐force intensity may preactivate primary and secondary sensory regions involved in processing the perceptual visual feedback resulting from its application, which, in turn, recruits the motor movements responsible for achieving the intended outcome. Thus, the EBA may facilitate the anticipation of motor outcomes and the multimodal representation of motor goals by linking sensory expectations to movement planning. This interpretation is further supported by recent fMRI MVPA studies, which show that EBA activation patterns reflect combinations of the intended goal, motor‐specific actions, and anticipated outcomes (Monaco et al. 2020; Rens et al. 2023).

In the light of these findings, it is likely that in our fMRI study the LOTC, and most likely the EBA, together with the IPS, supports an early stage of motor planning, in which the intended motor goal is represented in an effector‐specific, motor‐relevant reference frame (i.e., in body‐centered coordinates; Gallivan and Culham 2015) prior to movement preparation.

### 4.3 The Second Delay Period: Motor Preparation and Grip‐Force Anticipation in the M1

During the second delay period, our decoding analyses revealed lateralized clusters in the contralateral M1, within the lateral hand regions of Brodmann area 4 (BA4). These sub‐regions are well‐established for their role in movement preparation and execution (Rizzolatti et al. 1996; Picard and Strick 1996; Davare et al. 2009; Cisek and Kalaska 2010). In particular, neuroimaging, TMS, and clinical case studies have consistently shown that brain activation patterns underlying motor execution, as well as movement planning and preparation for left‐hand movements in right‐handed individuals, are similarly lateralized to the contralateral hemisphere, as compared to preparation for right‐hand movements (Dassonville et al. 1997; Klein et al. 2016).

While motor planning and preparation have been traditionally attributed to premotor and parietal brain regions (Ruiz et al. 2024), recent fMRI studies have demonstrated increased activation and above‐chance decoding in the contralateral M1 prior to movement execution, suggesting that the M1 might play a key role in preparing upcoming motor actions (Gale et al. 2021). These findings are consistent with earlier neurophysiological (Tanji and Evarts 1976; Riehle and Requin 1989) and univariate fMRI studies (Tanji and Evarts 1976), which consistently indicated that anticipatory activity in the contralateral M1 reflects movement intentions. More recently, fMRI MVPA investigations (Ariani et al. 2018, 2022; Gale et al. 2021) have further emphasized the involvement of the contralateral M1 in motor planning, showing that multivariate activation patterns in this region can predict planned finger movements, and movement sequences during a delay period. Time‐resolved MVPA has also revealed significant similarities in brain activation patterns underlying delayed and immediate movement plans (Ariani et al. 2018), suggesting that similar motor codes were used for both planning, and executing movements (Caccialupi et al. 2025). Together, these findings underscore the critical role of the M1 in encoding and planning detailed motor movements prior to execution.

Our results are therefore in line with findings from recent fMRI MVPA studies on movement planning; however, they slightly diverge from those of our previous work (Caccialupi et al. 2025). In that study, we used a delayed grip‐force task involving a single effector (right hand) and one 9‐s delay period, finding above‐chance decoding in the l‐PMd, but not in the M1. In contrast, the current experiment involved two 6‐s delay periods, explicitly distinguished by the initial presentation of effector cues, which required participants to prepare movements with either their left or right hand. The inclusion of two potential effectors (including the non‐dominant hand) and a shorter second delay period (as compared to the previous study) likely increased task complexity, as participants needed to first select the effector and then readily prepare the grip‐force execution. This increase in complexity likely prompted participants to begin preparing the grip‐force earlier than in the previous experiment. Once participants received the second‐effector cue, they were able to initiate grip‐force preparation, knowing that the task involved only two delay periods, and they had all the necessary information to set the motor parameters for execution. The explicit differentiation of delay phases might have provided stronger expectations for the upcoming task, thereby enhancing motor preparation. As indicated in previous studies, the structure of the delay period—particularly when divided into distinct phases—can modulate motor readiness and cortical engagement, especially within the M1 (Davare et al. 2009; Cisek and Kalaska 2010). Thus, the two delay periods, combined with the potential switch in hand preparation, likely induced more active and precise motor preparation in the M1s in the current experiment, as compared to the single‐phase delay in the previous study. While the previous study demonstrated movement planning, with the selection of graded movement parameters such as force intensity (Mizuguchi et al. 2013; Caccialupi et al. 2025), the increased task complexity and clearer temporal structure in the current design likely prompted participants to initiate motor preparation during the second delay period. The contribution of the M1, which is well known to‐be directly involved in generating motor output and pre‐activating muscle fibres (Dechent et al. 2004; Mizuguchi et al. 2013; Moreno et al. 2025), thus likely reflect motor preparation in the current study, leading to above‐chance decoding in the M1 as the task approach execution. The differences between the two studies may be ultimately attributed to distinct task demands (Caccialupi et al. 2025).

Taken together, above‐chance decoding in contralateral M1s during the second delay period, and systematic lateralization, indicate effector‐specific coding of grip‐force intensity anticipation during movement planning.

### 4.4 Cross‐Decoding: Effector‐Specific Representation of Movement Parameters

The cross‐decoding analyses provided further support for the effector‐specific representation of parametric grip‐force intensities, as indicated by the results of the main decoding analyses. Specifically, the *X‐Dec: Cross Hand* analyses did not reveal evidence for shared activation patterns, that is, the same or similar type of codes, when the SVR models were trained on one hand and tested on the other. The absence of shared parametric representations across effectors suggests that grip‐force intensities were not encoded in a hand‐independent format (Gallivan, McLean, Smith, and Culham 2011). If similar motor codes were used to prepare grip‐force with either hand, we would expect at least some degree of generalization in decoding performance across hands—yet this was not the case, even at a liberal threshold of *p* < 0.001 uncorrected or at an even more liberal threshold of *p* = 0.01 uncorrected.

The findings of the main analyses were further corroborated by the *X‐Dec: Switch/no‐Switch* analyses, based on an independent FIR model. In this first‐level GLM, each combination of effector‐cue sequence (across the two delay periods) and grip‐force intensity was modelled as an independent regressor, accounting for differences in BOLD dynamics between conditions (i.e., trials in which preparation with the target hand in the second delay followed a switch vs. no switch in effector‐cue). Despite this modelling approach, parametric decoding of anticipated grip‐force intensities for each cued hand resulted interestingly in above‐chance performance, restricted to contralateral M1s during the second delay period. Moreover, *X‐Dec: Switch/no‐Switch* analyses revealed no above‐chance decoding during the first delay period, in which the SVR was trained and tested on beta estimates reflecting different hands. Since above‐chance in this analysis would have likely indicated the presence of similar brain‐activation patterns for the encoding of grip‐force intensities with the two hands (i.e., effector independent format representation of grip‐force intensity), our results indirectly corroborate effector‐specific representation of anticipated grip‐force, as found in the main decoding analyses. Consistently, our findings also show robust and systematic lateralized encoding of grip‐force intensity during motor preparation.

Taken together, the results from *X‐Dec: Cross Hand* and *X‐Dec: Switch/no‐Switch* analyses indicate that grip‐force representations are lateralized in effector‐specific brain regions. This finding undermines the hypothesis that anticipated grip‐force intensities are parametrically represented in an effector‐independent format, that is, represented by shared or similar codes across hands. Importantly, the observed effector‐specific coding may be influenced by task demands (Christophel et al. 2017). Specifically, having cued the effector at the onset of each delay period may have promoted effector‐specific coding of force intensities and limited the emergence of more abstract, effector‐independent representations. As proposed by influential WM models (e.g., Christophel et al. 2017), the format of neural representations can vary depending on task requirements. Similarly, during grip‐force planning, parametric codes of force intensity may be represented in different formats and regions depending on whether the effector must be selected at each delay period or is fixed across conditions. In line with our previous study (Caccialupi et al. 2025), information about force intensity can be represented in effector‐independent (movement unspecific) codes within the vmPFC during force selection and later transformed into detailed motor codes when no effector choice is required. In contrast, because effector selection was required at the onset of every delay period in the present task, grip‐force intensities were likely encoded and prepared in distinct regions and representational formats across task phases, but consistently in an effector‐dependent manner. The present findings support the hypothesis that intended grip‐force intensities are first selected and represented in effector‐specific brain regions, and then transformed into motor codes, where movements are represented in a ready‐to‐use, effector‐specific format (Langner et al. 2014; Mylopoulos and Pacherie 2018; Van Ede and Nobre 2023; Caccialupi et al. 2025). The absence of shared coding across hands further argues against simultaneous representations of alternative motor plans, as posited by the affordance competition hypothesis (Cisek and Kalaska 2005) and previous EEG studies (Nasrawi et al. 2023) and instead aligns with a model of action planning that emphasizes sequential selection and transformation of intended actions into motor codes prior to execution (Van Ede 2020; Boettcher et al. 2021).

In conclusion, the cross‐decoding analyses provide converging evidence that information about anticipated grip‐force intensities, decoded from contralateral IPS, LOTCs, and M1, reflects the preparation of a specific motor plan for the selected effector, rather than effector‐independent representation or bilateral encoding of parametric grip‐force intensities.

### 4.5 Control Analyses

The specificity of the main decoding analyses is further corroborated by the results of three control analyses. First, label‐permutation testing demonstrates that prediction accuracies of the SVR decoding are based on the parametric coding of grip‐force intensities. Specifically, the computation of parametric contrasts on prediction accuracy maps, which resulted from label permutation testing, strongly corroborates the results of the main analysis, as it shows very similar clusters across the four time periods (at *p* < 0.05 FWE corrected). Second, SVR analyses restricted to accurate trials were conducted to assess the potential impact of performance accuracy on MVPA results. These control analyses revealed activation patterns highly consistent with those significant in the main decoding analysis (at *p* < 0.05 FWE‐corrected), indicating that variability in performance accuracy is unlikely to have substantially influenced the SVR results. Finally, univariate analyses revealed no significant parametric activity modulation during the two delay periods, and only in contralateral M1s during motor execution (at *p* < 0.05, FWE‐corrected). These results make it rather unlikely that parametric effects found in the main MVPA analyses were primarily driven by univariate effects. Taken together, results of the control analyses and null findings during the cue period indicate a high specificity and sensitivity of the reported findings.

## 5 Conclusion

This study provides novel insights into how grip‐force intensities are encoded during action selection and motor preparation in a delayed grip‐force task. Our results demonstrate that intended grip‐force intensities are represented parametrically in effector‐specific brain regions. Specifically, we observed early encoding in the contralateral IPS and LOTCs during the first delay period, followed by transformation into a motor code in the contralateral M1s during the second delay, as participants prepared for grip‐force execution. These findings are consistent with a two‐stage model of action planning, in which early motor planning underlies above‐chance decoding in the contralateral IPS and LOTCs, and more detailed motor parameters are encoded and prepared in the M1 before execution.

Additionally, our cross‐decoding analyses suggest that grip‐force representations in the brain are not shared between effectors, reinforcing the idea that motor codes are lateralized to specific brain regions based on the selected effector. These results do not indicate effector‐independent representation of parametric grip‐force intensities and align with a framework where motor plans are selected and prepared in contralateral sensorimotor areas. Furthermore, the consistent lateralization of grip‐force encoding across brain regions underscores the critical role of sensorimotor areas, such as the IPS, LOTCs, and M1s, in transforming sensory information into motor output.

Taken together, our findings provide compelling evidence for the effector‐specific, parametric encoding of motor plans across distinct transformation processes: from the selection of the intended grip‐force intensity to movement planning, preparation and execution. These results advance our understanding of motor planning and the contribution of lateralized brain‐networks in encoding and maintaining detailed motor parameters prior to movement execution. Future research may explore whether and how similar encoding principles apply for the planning of different motor actions.

## Funding

This work was supported by the German Academic Exchange Service (DAAD). The funder had no role in study design, data collection and analysis, decision to publish, or preparation of the manuscript.

## Ethics Statement

Participants gave their written consent before proceeding with the experiment. The experimental design and materials were approved by the ethics committee at Freie Universität Berlin (003/2021, Berlin, Germany).

## Conflicts of Interest

The authors declare no conflicts of interest.

## Supporting information


**Figure S1:** (A) Results of parametric contrast over prediction accuracy maps from the label permutation tests (see Methods), during preparation with the left hand (upper display, green background) or the right hand (lower display, green background) are presented across the same time periods as in the main analysis: cue period (C), first delay period (D1), second delay period (D2) and motor execution period (ME). Above‐chance prediction accuracy clusters are displayed at *p* < 0.05, FWE‐corrected (see Table S1). No brain region exhibited above‐chance decoding during C. As in the main decoding analysis, during D1, contralateral clusters in IPS and the r‐LOTC were revealed. In the first delay also the l‐LOTC was found at *p* < 0.001 uncorrected. The results of the main analysis were corroborated also for D2, by showing contralateral M1s, and the right supramarginal gyrus (r‐AngG). Finally, t‐contrasts on the ME revealed contralateral M1s and S1s. (B) Time‐courses of prediction accuracies of the permutation tests. Prediction accuracy values were extracted from the peak voxels of the six most representative clusters found in the main analysis (see Figure 3 and Table 1). These clusters include: r‐IPS [*x* = 24, *y* = −58, *z* = 58], l‐IPS [*x* = 42, *y* = −72, *z* = 18], r‐LOTC [*x* = −16, *y* = −74, *z* = 48], l‐LOTC [*x* = −40, *y* = −64, *z* = 14], r‐M1 [*x* = 36, *y* = −20, *z* = 62] and l‐M1 [*x* = −40, *y* = −16, *z* = 54]. The time‐courses represents prediction accuracy values obtained by averaging of time‐bins in correspondence of the second half of each period, as done in the main analysis (for plotting time‐courses and performing the statistical testing). The divergence of the permutations from the linear order of grip‐force levels is expressed as distance in rank order. As expected, the divergence from the original order reduces the performance of the SVR.
**Table S1:** T‐contrast of the parametric permutation testing revealed regions that exhibited above‐chance prediction accuracy across the cue period, first and second delay periods, and motor execution period (as in the main analysis), at *p* < 0.05, FWE corrected. The l‐LOTC was found at a *p* < 0.001 uncorrected level.
**Figure S2:** A results of second‐level *t*‐tests of parametric effects of motor execution with the left (green) and right hand (blue) (i.e., on time‐bins t20–t22). First‐level contrasts were computed with parametric contrast weights within FIR models, following the Fechner correction performed in the main analysis. Significant parametrically modulated activity was found in the contralateral right M1 and left M1, shown at *p* < 0.05, FWE‐corrected (see Table S2).
**Table S2:** Regions that exhibit univariate parametric activity modulation during motor execution, revealed by a t‐contrast displayed at *p* < 0.05, FWE corrected.
**Figure S3:** A results of time‐resolved SVR analyses on accurate trials. To assess the impact of performance accuracy on SVR decoding, analyses were conducted on beta estimates reflecting accurate trials only. This resulted in the inclusion of a subset of participants from the main decoding analyses (*N* = 10; inclusion criterion: at least three accurate trials per condition per run after exclusion of inaccurate trials). A displays brain regions that parametrically code grip‐force intensities during the cue period (C), first delay period (D1), second delay period (D2), and motor execution period (ME). The upper (green) panel indicates preparation with the left hand, and the lower (blue) panel indicates preparation with the right hand. Brain regions with above‐chance prediction accuracy are revealed by t‐contrasts testing the respective periods against zero, at *p* < 0.05, FWE corrected at the voxel level. As expected, no brain region exhibited above‐chance decoding during C (first column). In D1, two lateralized clusters were revealed in the IPS and LOTC on contralateral hemispheres (r‐IPS and l‐LOTC were significant only at *p* < 0.001, uncorrected, and are not displayed; see Table S3). In D2, contralateral M1 activity was observed (third column), indicating transformation of neural codes prior to execution. Finally, t‐contrasts during ME revealed contralateral and ipsilateral clusters in the M1s (fourth column). For this subset of participants (*N* = 10), analyses restricted to accurate trials yielded results consistent with those obtained using all trials, identifying the same regions with above‐chance decoding. Compared to the main decoding analysis (*N* = 25; see Figure 3 and Table 1), the same regions were observed at *p* < 0.05 FWE‐corrected, with the only exceptions being that, for the right hand (Delay 1), the l‐LOTC was found only at *p* < 0.001 uncorrected, and for the left hand (Delay 1), the r‐IPS was found only at *p* < 0.001 uncorrected. Together, these results suggest that variability in performance accuracy is unlikely to have influenced the SVR results in the main decoding analysis.
**Table S3:** Regions that exhibit above‐chance prediction accuracy across the cue period, first and second delay periods and motor execution period, revealed by a t‐contrast displayed at *p* < 0.05, FWE corrected at the voxel level. The r‐IPS and the l‐LOTC were only found at a *p* < 0.001 uncorrected level. Significant effects reflect trials with accurate grip‐force performance for a subset of participants from the main decoding analyses (*N* = 10).

## Data Availability

The data that support the findings of this study are available on request from the corresponding author. The data are not publicly available due to privacy or ethical restrictions.
