# Supplementary material for: Decoding Effector‐Specific Parametric Grip‐Force Anticipation From fMRI‐Data
Source: Hum Brain Mapp. 2025 Dec 30;47(1):e70441. doi: 10.1002/hbm.70441 (PMC12753588; doi:10.1002/hbm.70441)
Supplement: Supplementary file 1 — Figure S1: (A) Results of parametric contrast over prediction accuracy maps from the label permutation tests (see Methods), during preparation with the left hand (upper display, green background) or the right hand (lower display, green background) are presented across the same time periods as in the main analysis: cue period (C), first delay period (D1), second delay period (D2) and motor execution period (ME). Above‐chance prediction accuracy clusters are displayed at p < 0.05, FWE‐corrected (see Table S1). No brain region exhibited above‐chance decoding during C. As in the main decoding analysis, during D1, contralateral clusters in IPS and the r‐LOTC were revealed. In the first delay also the l‐LOTC was found at p < 0.001 uncorrected. The results of the main analysis were corroborated also for D2, by showing contralateral M1s, and the right supramarginal gyrus (r‐AngG). Finally, t‐contrasts on the ME revealed contralateral M1s and S1s. (B) Time‐courses of prediction accuracies of the permutation tests. Prediction accuracy values were extracted from the peak voxels of the six most representative clusters found in the main analysis (see Figure 3 and Table 1). These clusters include: r‐IPS [x = 24, y = −58, z = 58], l‐IPS [x = 42, y = −72, z = 18], r‐LOTC [x = −16, y = −74, z = 48], l‐LOTC [x = −40, y = −64, z = 14], r‐M1 [x = 36, y = −20, z = 62] and l‐M1 [x = −40, y = −16, z = 54]. The time‐courses represents prediction accuracy values obtained by averaging of time‐bins in correspondence of the second half of each period, as done in the main analysis (for plotting time‐courses and performing the statistical testing). The divergence of the permutations from the linear order of grip‐force levels is expressed as distance in rank order. As expected, the divergence from the original order reduces the performance of the SVR. Table S1: T‐contrast of the parametric permutation testing revealed regions that exhibited above‐chance prediction accuracy across the cue period, [file HBM-47-e70441-s001.docx]

**Decoding Effector-Specific Parametric Grip-Force Anticipation from fMRI-Data: Supplementary materials**


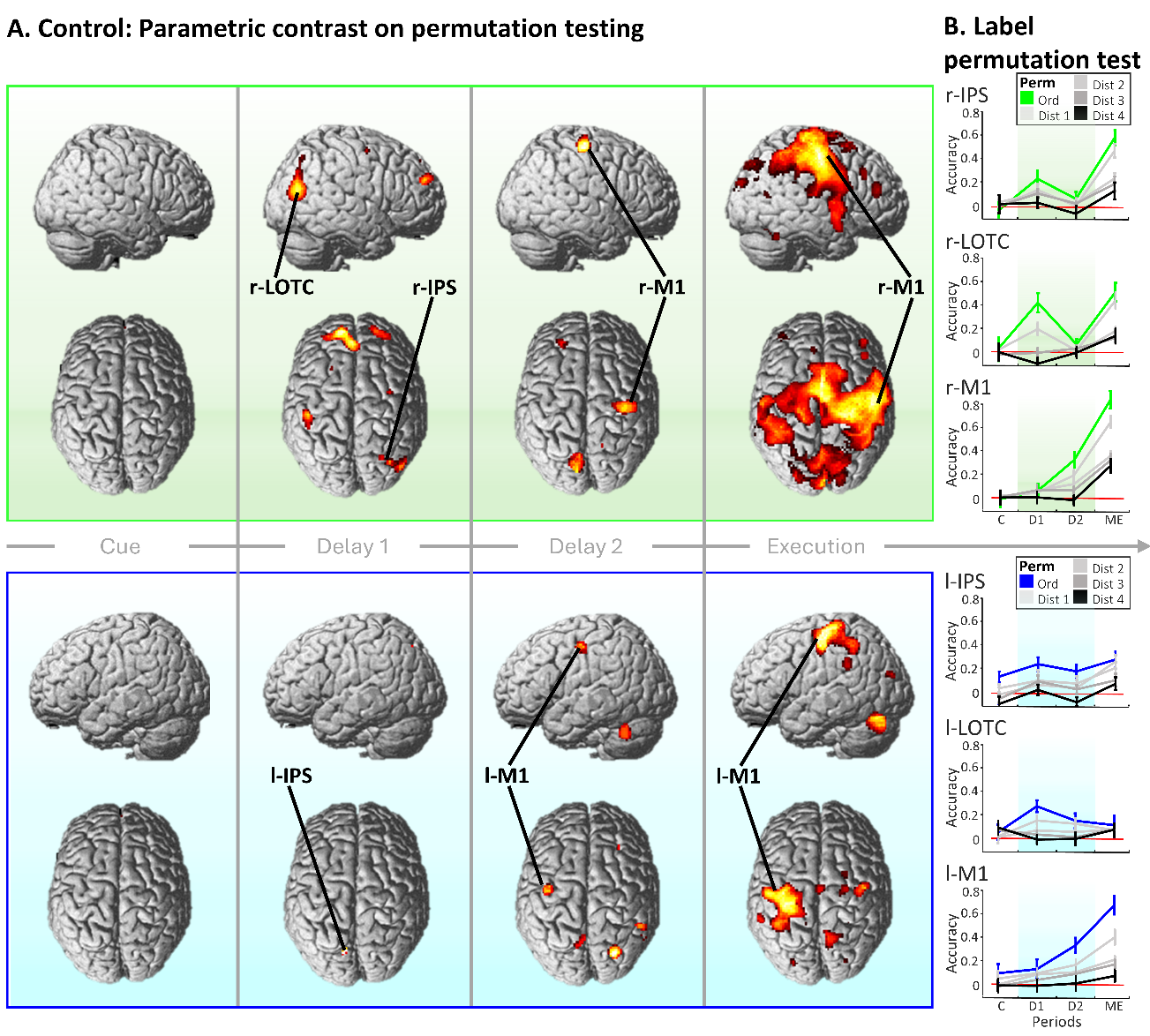


**Figure S1 A.** Results of parametric contrast over prediction accuracy maps from the label permutation tests (see Methods), during preparation with the left hand (upper display, green background) or the right hand (lower display, green background) are presented across the same time periods as in the main analysis: cue period (C), first delay period (D1), second delay period (D2) and motor execution period (ME). Above-chance prediction accuracy clusters are displayed at *p* < 0.05, FWE-corrected (see Table S1). No brain region exhibited above-chance decoding during C. As in the main decoding analysis, during D1, contralateral clusters in IPS and the r-LOTC were revealed. In the first delay also the l-LOTC was found at *p* < 0.001 uncorrected. The results of the main analysis were corroborated also for D2, by showing contralateral M1s, and the right supramarginal gyrus (r-AngG). Finally, t-contrasts on the ME revealed contralateral M1s and S1s. **B.** Time-courses of prediction accuracies of the permutation tests. Prediction accuracy values were extracted from the peak voxels of the six most representative clusters found in the main analysis (see Figure 3 and Table 1). These clusters include: r-IPS [x=24, y=--58, z=58], l-IPS [x=42, y=-72, z=18], r-LOTC [x=-16, y=-74, z=48], l-LOTC [x=-40, y=-64, z=14], r-M1 [x=36, y=-20, z=62] and l-M1 [x=-40, y=-16, z=54]. The time-courses represents prediction accuracy values obtained by averaging of time-bins in correspondence of the second half of each period, as done in the main analysis (for plotting time-courses and performing the statistical testing). The divergence of the permutations from the linear order of grip-force levels is expressed as distance in rank order. As expected, the divergence from the original order reduces the performance of the SVR.

| Cluster size | Anatomical Region | Peak MNI coordinates | | |  |
| --- | --- | --- | --- | --- | --- |
|  |  | x | y | z | z-score |
| **First delay Period, left hand** | | | | | |
| 596 | Right LOTC | 42 | -74 | 18 | 7.00 |
|  | Right IPS | 26 | -68 | 48 | 4.70 |
| 723 | Left MSFG | -2 | 42 | 38 | 5.59 |
| 165 | Right SFG | 20 | 56 | 30 | 5.15 |
| 180 | Left S1 | 38 | 54 | 20 | 5.05 |
| 16 | Right PMd | 34 | -4 | 56 | 4.71 |
| **First delay Period, right hand** | | | | | |
| 19 | Left IPS | -12 | -72 | 50 | 4.72 |
| Additional cluster in the Left LOTC at *p* < 0.001 | | | | | |
| 54 | Left LOTC | -40 | -64 | 14 | 3.59 |
| **Second delay Period, left hand** | | | | | |
| 323 | Right M1 | 38 | -20 | 62 | 6.16 |
| 300 | Left IPS | -10 | -76 | 44 | 5.89 |
| 6 | Left MFG | -22 | 38 | 30 | 5.19 |
| **Second delay Period, right hand** | | | | | |
| 118 | Left M1 | -40 | -14 | 48 | 5.84 |
| 185 | Right SPL | 26 | -74 | 52 | 5.61 |
| 125 | Left cerebellum | -46 | -56 | -35 | 5.41 |
| 77 | Left IPS | -8 | -62 | 50 | 5.10 |
|  | Right AngG | 52 | -60 | 20 | 4.88 |
| 12 | Right MFG | 30 | 28 | 36 | 4.77 |
| **Motor execution Period, left hand** | | | | | |
| 27690 | Right M1 | 24 | -24 | 62 | 9.95 |
| 114 | Right MFG | 40 | 42 | 18 | 5.25 |
| 160 | Left MFG | -36 | 46 | 10 | 5.15 |
| **Motor execution Period, right hand** | | | | | |
| 2553 | Left M1 | -34 | -20 | 54 | 6.79 |
| 1572 | Left cerebellum | -34 | -72 | 26 | 6.69 |
| 199 | Right M1 | 40 | -14 | 62 | 5.28 |
| 252 | Right IPS | 12 | -62 | 60 | 5.16 |
| 70 | SMA | 0 | -14 | 56 | 5.01 |
| 74 | Left SMG | -58 | 42 | 30 | 4.80 |

**Table S1** T-contrast of the parametric permutation testing revealed regions that exhibited above-chance prediction accuracy across the cue period, first and second delay periods, and motor execution period (as in the main analysis), at *p* < 0.05, FWE corrected. The l-LOTC was found at a p < 0. 001 uncorrected level.


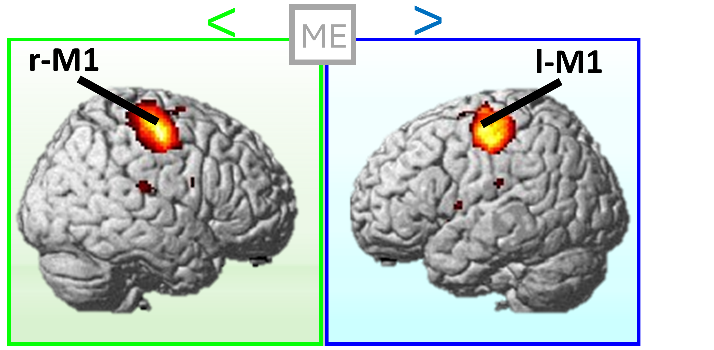


**Figure S2 A.** Results of second-level t-tests of parametric effects of motor execution with the left (green) and right hand (blue) (i.e., on time-bins t20-t22). First-level contrasts were computed with parametric contrast weights within FIR models, following the Fechner correction performed in the main analysis. Significant parametrically modulated activity was found in the contralateral right M1 and left M1, shown at p < 0.05, FWE-corrected (see Table S2).

| **Motor execution Period, left hand** | | | | | |
| --- | --- | --- | --- | --- | --- |
| 4468 | Right M1 | 40 | -18 | 54 | 10.03 |
| **Motor execution Period, right hand** | | | | | |
| 3646 | Left M1 | -38 | -20 | 54 | 10.10 |

**Table S2** Regions that exhibit univariate parametric activity modulation during motor execution, revealed by a t-contrast displayed at p < 0.05, FWE corrected.


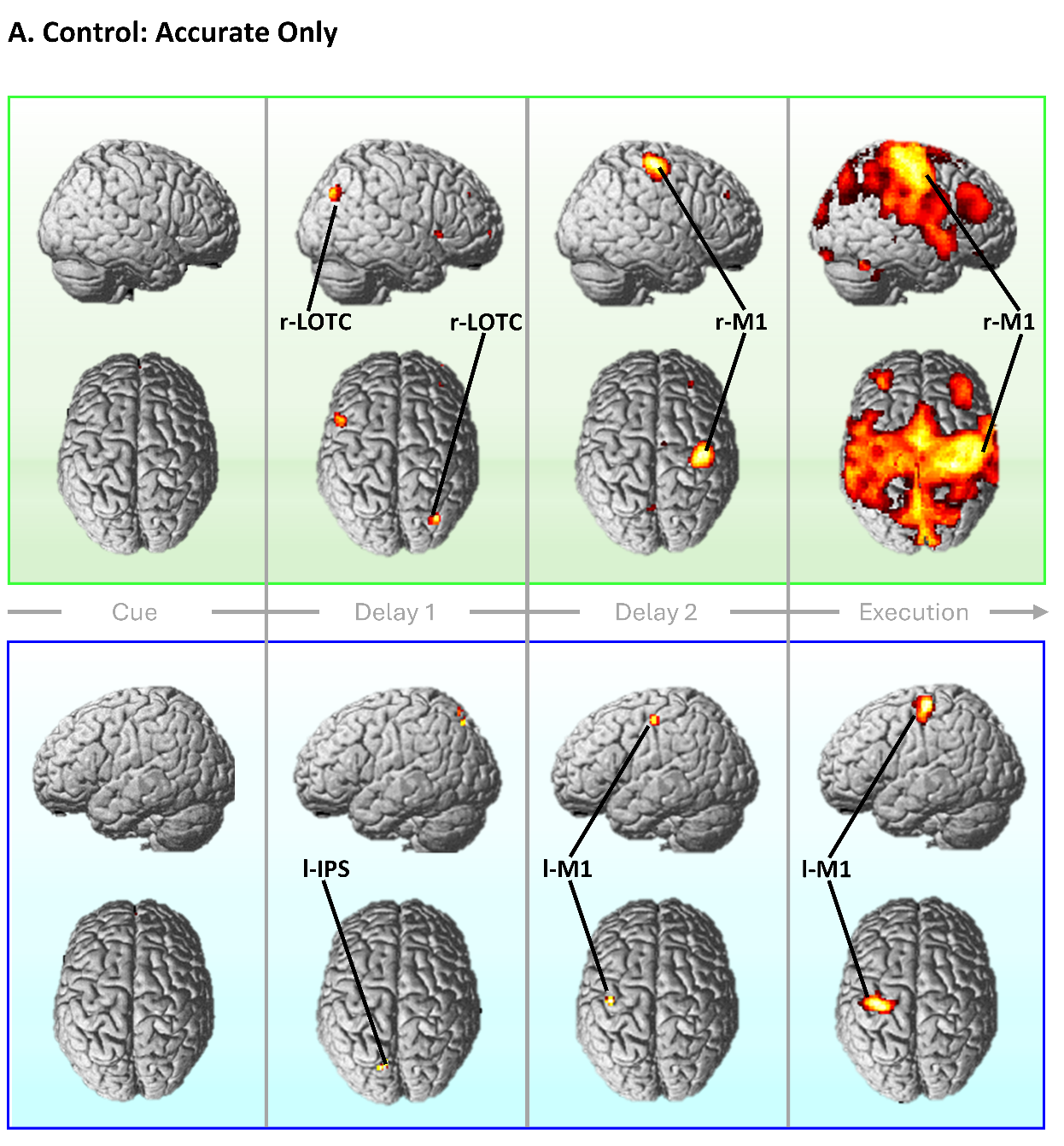


**Figure S3 A.** Results of time-resolved SVR analyses on accurate trials. To assess the impact of performance accuracy on SVR decoding, analyses were conducted on beta estimates reflecting accurate trials only. This resulted in the inclusion of a subset of participants from the main decoding analyses (*N* = 10; inclusion criterion: at least three accurate trials per condition per run after exclusion of inaccurate trials). **A.** Displays brain regions that parametrically code grip-force intensities during the cue period (C), first delay period (D1), second delay period (D2), and motor execution period (ME). The upper (green) panel indicates preparation with the left hand, and the lower (blue) panel indicates preparation with the right hand. Brain regions with above-chance prediction accuracy are revealed by t-contrasts testing the respective periods against zero, at *p* < 0.05, FWE corrected at the voxel level. As expected, no brain region exhibited above-chance decoding during C (first column). In D1, two lateralized clusters were revealed in the IPS and LOTC on contralateral hemispheres (r-IPS and l-LOTC were significant only at *p* < 0.001, uncorrected, and are not displayed; see Table S3). In D2, contralateral M1 activity was observed (third column), indicating transformation of neural codes prior to execution. Finally, t-contrasts during ME revealed contralateral and ipsilateral clusters in the M1s (fourth column). For this subset of participants (*N* = 10), analyses restricted to accurate trials yielded results consistent with those obtained using all trials, identifying the same regions with above-chance decoding. Compared to the main decoding analysis (*N* = 25; see Figure 3 and Table 1), the same regions were observed at *p* < 0.05 FWE-corrected, with the only exceptions being that, for the right hand (Delay 1), the l-LOTC was found only at *p* < 0.001 uncorrected, and for the left hand (Delay 1), the r-IPS was found only at *p* < 0.001 uncorrected. Together, these results suggest that variability in performance accuracy is unlikely to have influenced the SVR results in the main decoding analysis.

| Cluster size | Anatomical Region | Peak MNI coordinates | | |  |
| --- | --- | --- | --- | --- | --- |
|  |  | x | y | z | z-score |
| **First delay Period, left hand** | | | | | |
| 147 | Right LOTC | 32 | -72 | 36 | 5.55 |
| 101 | Left MFG | -50 | 10 | 34 | 5.05 |
| Additional cluster in the Right IPS at *p* < 0.001 | | | | | |
| 14 | Right IPS | 6 | -58 | 60 | 4.32 |
| **First delay Period, right hand** | | | | | |
| 21 | Left IPS | -16 | -74 | 50 | 4.78 |
| Additional cluster in the Left LOTC at *p* < 0.001 | | | | | |
| 102 | Left LOTC | -26 | -70 | 18 | 3.78 |
| **Second delay Period, left hand** | | | | | |
| 810 | Right M1 | 38 | -18 | 62 | 6.25 |
| **Second delay Period, right hand** | | | | | |
| 50 | Left M1 | -42 | -18 | 54 | 5.15 |
| **Motor execution Period, left hand** | | | | | |
| 43803 | Right M1 | 30 | -24 | 58 | 9.68 |
| 818 | Right MFG | 38 | 48 | 18 | 6.15 |
| 573 | Left MFG | -28 | 46 | 22 | 6.14 |
| **Motor execution Period, right hand** | | | | | |
| 637 | Left M1 | -28 | -24 | 60 | 5.65 |

**Table S3** Regions that exhibit above-chance prediction accuracy across the cue period, first and second delay periods and motor execution period, revealed by a t-contrast displayed at *p* < 0.05, FWE corrected at the voxel level. The r-IPS and the l-LOTC were only found at a *p* < 0. 001 uncorrected level. Significant effects reflect trials with accurate grip-force performance for a subset of participants from the main decoding analyses (*N* = 10).
